# The DOTA macrocyclic cavity in metallic radiopharmaceuticals: Mythology or reality?

**DOI:** 10.1186/s41181-023-00202-6

**Published:** 2023-08-03

**Authors:** Adriano Duatti

**Affiliations:** Department of Chemical and Pharmaceutical Sciences, University of Ferrara, Via L. Borsari, 46, 44121 Ferrara, Italy

**Keywords:** DOTA, Macrocyclic cavity, Macrocyclic ligands, Macrocyclic effect, Metallic radiopharmaceuticals

## Abstract

**Background:**

The hypothetical concept of ‘macrocyclic cavity’ is largely employed as useful model to interpret the affinity of metal ions for the macrocyclic chelating ligand 2,2′,2′′,2′′′-(1,4,7,10-tetraazacyclododecane-1,4,7,10-tetrayl)tetraacetic acid (H_4_DOTA). It Is hypothesized that a close matching between the size of the macrocyclic cavity and that of the metallic ion is a key parameter to ensure the high-yield formation of stable coordination metal-DOTA complex. This approach has become popular in the design of radiopharmaceuticals containing radiometals and H_4_DOTA as chelating group.

**Results:**

Based on X-ray structural data of metallic complexes formed by the ligand H_4_DOTA upon coordination with a variety of metals, an elementary argument based on Euclidean geometry is presented here that questions the existence of the hypothetical ‘macrocyclic cavity’ within the chelator macrocycle. The geometrical analysis was applied to the complex formed by a Ga^3+^ ion coordinated to H_4_DOTA as model compound.

**Conclusions:**

Application of Euclidean geometry to calculate bond angles in the coordination complex of the ligand H_4_DOTA with the Ga^+3^ ion, supposed to incorporate a hypothetical ‘macrocyclic cavity’, revealed that this conceptual entity has no physical reality and, therefore, cannot be considered a meaningful description of a stable structural arrangement for metallic radiopharmaceuticals.

## Background

The notion of ‘macrocyclic cavity’ has become popular after Pedersen’s discovery of crown ethers in 1967 and has been used as a key theoretical hypothesis to explain the strong affinity of this class of macrocyclic ligands for alkali metal ions (Pedersen 1967). Since the outer electron distribution of an alkali metal ion always overlaps that of the noble gas that precedes it in the periodic table, the inherent inertness of this closed-shell electronic configuration prevents the formation of strong covalent bonds between the ligand and the cation. Therefore, to account for the high selectivity of crown ethers for alkali metal ions the model of ‘macrocyclic cavity’ was introduced. It has been argued that a stable coordination complex between the alkali metal cation and the crown ether can be formed when there exists an appropriate match between the size of the ion and the cavity size of the crown ether (Liou and Brodbelt 1992). In this example, the size of the cation is simply interpreted as its volume calculated from the value of its ionic radius, whereas the size of the macrocyclic cavity is conventionally determined by considering the bond distances of the main atoms surrounding the cavity and drawing its boundaries, as measured from X-ray diffraction data (Henrick et al. 1984; Henrick et al. 2007; Comba 1999; McNeil et al. 2022).

After its introduction in radiopharmaceutical chemistry, the macrocyclic ligand 2,2′,2",2‴-(1,4,7,10-tetraazacyclododecane-1,4,7,10-tetrayl)tetraacetic acid (H_4_DOTA) has been popularized as a kind of ‘universal’ polymetallic chelating agent (Stasiuk and Long 2013; Baranyai et al. 2020) able to form stable complexes with a multitude of metallic cations. This hypothetical universal coordination affinity of the DOTA ligand towards radiometals was initially rationalized by considering quantities such as the thermodynamic stability constant, the macrocyclic effect, and the strength of the metal-nitrogen bonds. However, the idea that a cationic radiometal binds H_4_DOTA based on size fit with the interior of a hypothetical cavity associated with the macrocyclic ring has progressively gained widespread acceptance and, as a result, is often applied to the design of metallic radiopharmaceuticals particularly with the new emerging rare-earth radiometals (Stasiuk and Long 2013; Baranyai et al. 2020; Hancock 1992; Hancock and Martell 1989). Still, it is reasonable to ask whether this cavity really exists. When forming a metallic complex with H_4_DOTA, do cationic radiometals truly enter this sort of virtual hole lurking within the macrocyclic ligand the same way alkali metal ions are trapped within crown ethers?

Although in the past years, various authors (Hancock 1992; Hancock and Martell 1989) had questioned the concept of size-match selectivity between the dimensions of the macrocyclic cavity and the radius of the metallic ion, in the radiopharmaceutical literature this intuitive and easy to understand model continues to be extensively utilized despite the lack of a scientifically rigorous analysis of its structural nature and real existence.

The purpose of this study is to present a critical review of the concept of ‘macrocyclic cavity’ applied to the design and preparation of metallic radiopharmaceuticals with the ligand H_4_DOTA. The focus on H_4_DOTA constitutes an obvious choice because of the role of ‘universal chelating system’ attributed to this macrocycle, a property mostly inferred from a comparison of the size of the radiometal ion and that of the putative DOTA macrocyclic cavity (Henrick et al. 2007; Stasiuk and Long 2013; Baranyai et al. 2020; Hancock 1992; Hancock and Martell 1989; Urbanovský et al. 2020). Following this vague and unrealistic speculation, the radiopharmaceutical community has naively used H_4_DOTA with all radiometals and, specifically, with the so-called ‘trivalent ions’ such as Ga^3+^, Lu^3+^ Y^3+^, Ac^3+^ and Bi^3+^. It is worthy to note that the terminology ‘trivalent ion’, widely used to indicate a cation with a + 3 net charge, is not consistent with the standard definition of ‘valency’ given in the *Compendium of Chemical Terminology*, which implies no logical relationship to the notion of ionic charge (Compendium of Chemical Terminology (Gold Book) 2014).

The concept of ‘macrocyclic ligand’ is closely related to that of ‘chelating ligand’ (Hancock and Martell 1989). Both types of ligands bind a metallic center with multiple coordinating atoms linked together by chemical bonds to form a polydentate ligand. In contrast, a monodentate ligand binds the metal atom through only one coordinating atom. When the chain of coordinating atoms in a polydentate ligand forms a closed loop, this gives rise to a ‘macrocyclic ligand’. It is exactly the size of this internal ring that inspired the concept of ‘macrocyclic cavity’.

The ‘macrocyclic chelate effect’ refers to the increase in thermodynamic stabilization of the metal complex when *n* monodentate ligands are replaced by a macrocyclic ligand containing the same *n* coordinating atoms connected in a ring (Hancock 1992; Hancock and Martell 1989). It is considered essentially an entropic effect generated by the decrease of free energy of the reaction of complex formation between the metal and the macrocycle brought about by the concomitant decrease of the entropy of reaction according to the equation ΔG° = ΔH° – TΔS° (where ΔG° is the change of standard free energy, ΔH° is the change in standard enthalpy, T is the absolute temperature and ΔS° is the change in standard entropy). Since the enthalpic term ΔH° is associated with the formation of new chemical bonds, its impact is considered negligible because the metal forms the same type of bond with a coordinating atom whatever it belongs to a monodentate or macrocyclic chelating ligand. Therefore, it is the entropic term that apparently governs the stabilization of the macrocyclic complex and a decrease in entropy leads to the favorable decrease of free energy. Among the entropic effects that are commonly held responsible for the decrease in entropy the close matching between the size of the metal ion and that of the hypothetical macrocyclic cavity is also included. As discussed below, the notion of macrocyclic cavity is not rigorously defined and, moreover, it is not even necessary to explain the experimental facts. Instead, it will be argued that it is precisely the metal electronic properties that determine the observed chemical behavior (Hancock 1992; Hancock and Martell 1989) and that the entropic factor associated with the model of the hypothetical macrocyclic cavity is largely redundant.

## Methods

The simple geometrical model illustrated below has been used to analyze and question the concept of ‘macrocyclic cavity’.

**Fig. 1:**
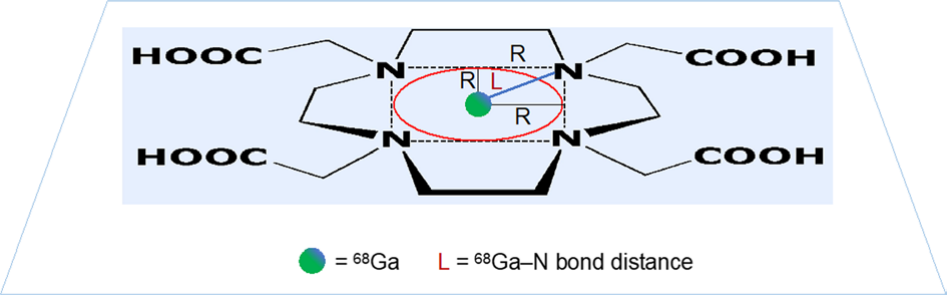
Idealized drawing of the structure of the H_4_DOTA ligand and of the location of the hypothetical ‘macrocyclic cavity’ assumed to be of spherical shape (not shown) and positioned at the center of the N4 plane. The red line is the circumference generated by the intersection of the sphere with the N4 plane

Figure 1 shows an idealized drawing of the chemical structure of the H_4_DOTA ligand (Stasiuk and Long 2013) and the position of the hypothetical spherical cavity at the center of the plane defined by the four coordinating nitrogen atoms. The circle marked in red is generated by the intersection of this plane with the surface of the spherical cavity along the equatorial line.

**Fig. 2:**
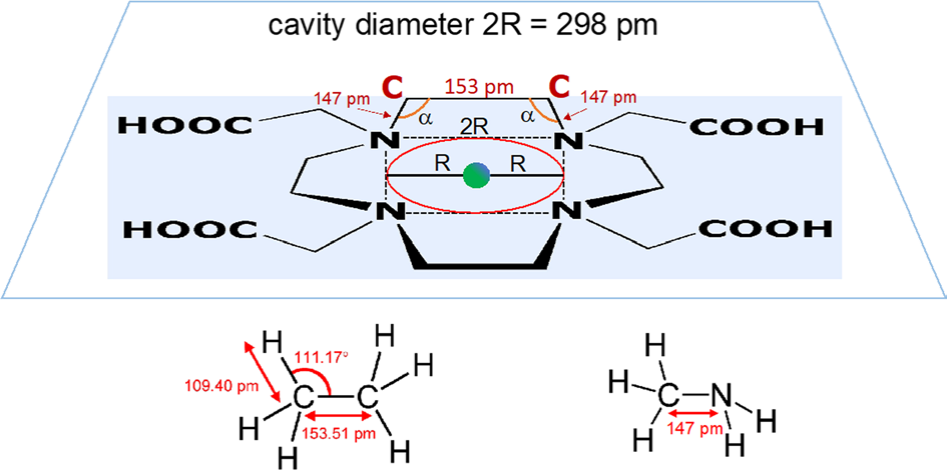
Idealized drawing of the coordination of a Ga^3+^ ion inside the hypothetical spherical cavity (not shown) located at the center of the plane defined by the four coordinated nitrogen atoms. L is the Ga–N bond distance, R is the radius of the cavity, and the red circle is the circumference generated by the intersection of the spherical hole with the N4 plane

To better illustrate this model, it is convenient to imagine that the DOTA macrocycle is coordinated to a Ga^3+^ ion as exemplified by a large number of Ga-DOTA complexes and ^68^ Ga-DOTA radiopharmaceuticals (Viola et al. 2006; Davey and Paterson 2022). In Fig. 2, the green ball represents a Ga^+3^ ion placed in the center of the plane defined by the four nitrogen atoms of the H_4_DOTA ligand, R is the radius of the hypothetical spherical cavity, and L is the Ga–N bond distance (Pyykkö and Atsumi 2009).

## Results

**Fig. 3:**
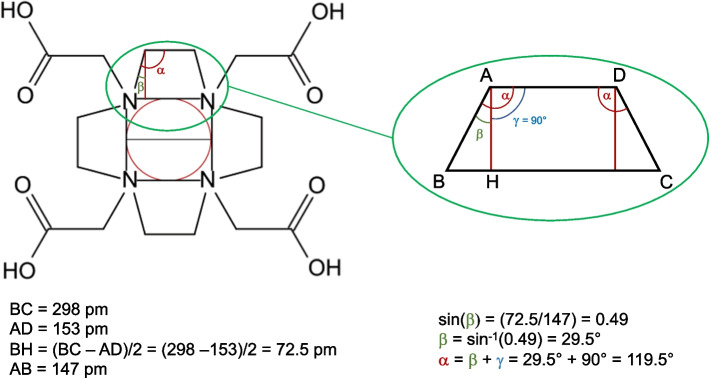
Calculations of the amplitude of N $\widehat{C}$ C angles of the ethyl bridges surrounding the hypothetical macrocyclic cavity of the H_4_DOTA ligand

A simple geometrical calculation carried out using the model illustrated in Fig. 2, reveals that this spherical hole cannot exists. By applying the Pythagorean theorem, the value of the square of the Ga–N bond length, L^2^, can be expressed as L^2^ = 2R^2^. Hence, R^2^ = L^2^/2 and R = √(L^2^/2). The observed, average Ga–N bond length, determined by X-ray crystallography, is 211 pm (Hancock 1992). Consequently, R = √[(211)^2^/2] = 149 pm. The resulting value of the diameter of the cavity is (2 × 149) pm = 298 pm. However, in the DOTA macrocycle, the pairs of adjacent nitrogen atoms are connected by ethyl bridges as shown in Fig. 3. In this organic functional group, the C–C bond distance is only 153.51 pm (Harmony 1990). To contain a spherical hole of diameter 298 pm, the two tetrahedral angles N $\widehat{\mathrm{C}}$ C and C $\widehat{\mathrm{C}}$ N of the ethyl groups (α in Fig. 3) should be stretched from their normal value of 111.17° to approximately 119.5°. Figure 3 reports how α can be easily calculated using simple geometrical formulas of elementary Euclidian geometry for determining the internal angles of a trapeze. Obviously, to stretch the two α angles from 111° to 120° is equivalent to transform the hybridization of C atoms from tetrahedral (*sp*^3^) to trigonal planar (*sp*^2^). If this perturbation of the electronic distribution of the DOTA chemical bonds were to actually occur, it inevitably leads to a dramatic destabilization and reorganization of the macrocyclic ligand.

## Discussion

**Fig. 4:**
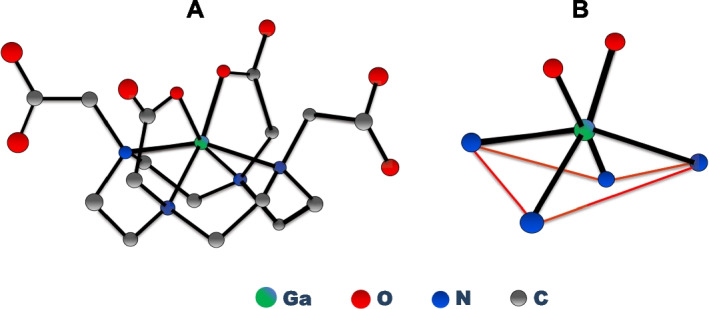
**A** Graphical illustration of the coordination arrangement in the complex [Ga(HDOTA)], and **B** of the geometrical arrangement of the four nitrogen donor atoms of DOTA around the Ga^3+^ ion. Drawings A and B have been created based on data reported in reference (Viola et al. 2006) and hydrogen atoms have been omitted for clarity

The geometrical analysis reported here easily explains the structural arrangement that the gallium atom and the DOTA ligand are forced to achieve because of the non-existence of the unnatural physical cavity in the complex [Ga(HDOTA)] (Viola et al. 2006). Single-crystal X-ray diffraction determination (Fig. 4A) of the structure of [Ga(HDOTA)] (Viola et al. 2006), showed that the Ga atom is not lodged in a cavity, but it is displaced out of the plane of the four nitrogen atoms, as illustrated in Fig. 4B. Evidently, it is hard to imagine the existence of a spherical cavity intersected by the N4 plane. Perhaps, the arrangement of the four nitrogen atoms could be best pictured as a pocket below the metal ion, or alternatively as a hat above the same ion. Whichever representation is chosen, this is the only structural arrangement that allows four Ga–N bonds of 211 pm to be accommodated within the same octahedral structure.

**Fig. 5:**
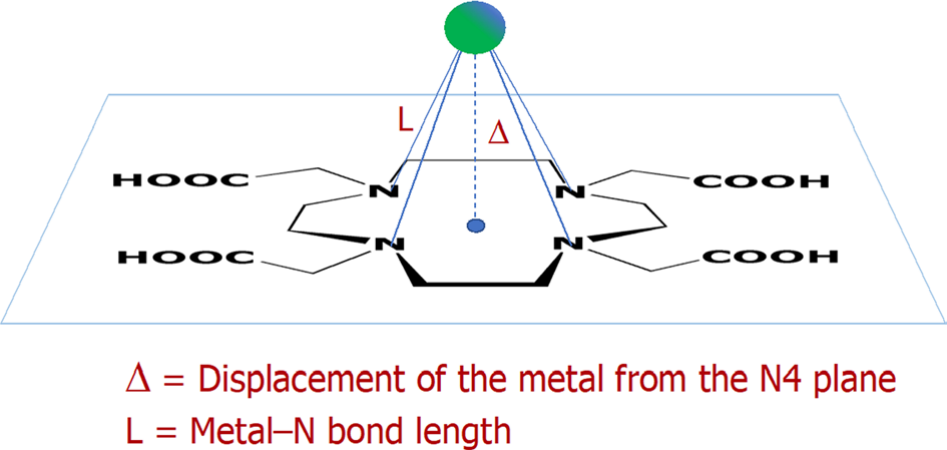
Pictorial representation of the location of the Ga^3+^ ion relative to the N4 plane in a generic Ga(DOTA) complex as determined by X-ray crystal data (Viola et al. 2006)

The structural features observed in the [Ga(HDOTA)] (Compendium of Chemical Terminology (Gold Book) 2014) complex are repeatedly found in almost all complexes that the DOTA ligand forms with common metal ions in the + 2 and + 3 oxidation states (Viola-Villegas and Doyle 2009). The geometrical model that can conveniently represent the properties of these DOTA complexes is shown in Fig. 5.

The parameter Δ is a measure of the displacement of the metal ion out of the N4 plane and, hence, a measurable physical quantity that unequivocally demonstrates that the concept of the DOTA ‘macrocyclic cavity’ is another glaringly obvious example of misconception in science. In fact, there are no examples of DOTA metal complexes with a zero value of Δ. This parameter can range from 84.0 pm in the six-coordinated Ga^3+^ complex {[Ga(HDOTA)]⋅5.5H_2_O} and 105.8 pm in the seven-coordinated Fe^3+^ complex {Na[Fe(DOTA)]⋅5H_2_O} to 158.6 pm and 161.6 pm in the nine-coordinated complexes {Na[M(DOTA)(H_2_O)]⋅4H_2_O} (M = Lu^3+^ and Y^3+^), respectively (Viola-Villegas and Doyle 2009).

The lesson that can be drawn from these observations is that electronic factors are always predominant in determining the structure of a metal complex and, in turn, of metallic radiopharmaceuticals. More precisely, it is the electronic configuration of the metal ion and the energy distribution and symmetry of molecular orbitals that shape the structure of a coordination compound. Electronic factors (*i.e.*, enthalpic) govern the formation of chemical bonds and establish their characteristic lengths that are almost completely independent of steric (*i.e.*, entropic) factors. Ultimately, electronic factors dictate the spatial arrangement of the ligands around the metallic center, thus controlling the final geometry of a metal complex. In short, it is the ligand that adapts to the metal and not the reverse. These considerations raise many questions about the real existence of a hypothetical cavity inside the DOTA macrocyclic ligand and question the widespread habit of designing the structure of a metallic radiopharmaceutical based on the matching between the size of the ion and the dimensions of the elusive cavity.

## Conclusions

This short review attempted to question the scientific rationale underlying the concept of ‘macrocyclic cavity’ which is widely employed to interpret the stability of metallic complexes and radiopharmaceuticals containing the DOTA chelator. Using the structural data collected with the single-crystal X-ray diffraction technique on many DOTA complexes with a variety of metals and elementary Euclidean geometry, it has been shown that the macrocyclic cavity model is not supported by rigorous scientific evidence. On the contrary, the structural data show that, in metal-DOTA complexes, the metal ion tends to move from the position where the macrocyclic cavity is supposed to exist (at the intersection with the plane of the four nitrogen atoms) and, consequently, the DOTA ligand must undergo some deformations to adapt its conformation to the requirements imposed by the characteristic length of the metal-nitrogen chemical bonds. The need to distort the structure of the DOTA ligand is also revealed by the fact that radiolabeling reactions always requires heating to obtain a satisfactory radiochemical yield. If analyzed together, these observations lead to the conclusion that it is not scientifically justified to assume that in complexes with the DOTA ligand it is the metal that must adapt to the conformation of the hypothetical macrocyclic cavity, but rather the exact opposite occurs, and it is the DOTA ligand that undergoes a rearrangement to meet the electronic requirements of the metal. This change of perspective could prove useful for a more reliable assessment of the stability of radiopharmaceuticals containing the DOTA ligand.

## Data Availability

Not applicable.

## Abbreviation

H_4_DOTA: 2,2′,2′′,2′′′-(1,4,7,10-Tetraazacyclododecane-1,4,7,10-tetrayl)tetraacetic acid
